# Monitoring and assessment of water health quality in the Tajan River, Iran using physicochemical, fish and macroinvertebrates indices

**DOI:** 10.1186/s40201-015-0186-y

**Published:** 2015-04-16

**Authors:** Jaber Aazami, Abbas Esmaili-Sari, Asghar Abdoli, Hormoz Sohrabi, Paul J Van den Brink

**Affiliations:** Department of Environment, Faculty of Natural Resources, Tarbiat Modares University, Tehran, Iran; Department of Biodiversity and Ecosystem Management, Environmental Research Institute, Shahid Beheshti University, Tehran, Iran; Department of Forestry, Faculty of Natural Resources, Tarbiat Modares University, Tehran, Iran; Department of Aquatic Ecology and Water Quality Management, Wageningen University, Wageningen University and Research Centre, Wageningen, The Netherlands; Alterra, Wageningen University and Research Centre, Wageningen, The Netherlands

**Keywords:** Water health quality, Bio-indicators, Tajan, Physicochemical parameters

## Abstract

**Background:**

Nowadays, aquatic organisms are used as bio-indicators to assess ecological water quality in western regions, but have hardly been used in an Iranian context. We, therefore, evaluated the suitability of several indices to assess the water quality for an Iranian case study.

**Methods:**

Measured data on biotic (fish and macroinvertebrates) and abiotic elements (28 physicochemical and habitat parameters), were used to calculate six indices for assessment of water quality and the impact of human activities in the Tajan river, Iran. GIS, uni- and multivariate statistics were used to assess the correlations between biological and environmental endpoints.

**Results:**

The results showed that ecological condition and water quality were reduced from up- to downstream. The reduced water quality was revealed by the biotic indices better than the abiotic ones which were linked to a variety of ecological water quality scales.

**Conclusion:**

The fish index showed a strong relationship with long-term database of physicochemical parameters (12 years (94%)), whereas macroinvertebrates index is more correlated with short-term data (76%). Meanwhile, the biotic and abiotic elements in this study were also classified well by PCA. Pulp and wood plants and sand mining are indicated to have the most negative effects on the river ecosystem.

## Introduction

One of the greatest environmental challenges of this century is to sustain natural biological structural and functional attributes of aquatic ecosystems, rivers in particular. This goal requires that we know the condition of these dynamic systems and how they are being affected by specific factors and forces [1]. Nowadays, we can easily see that there are many pollutants in the environment due to anthropogenic activities. The destruction of natural habitats and the presence of environmental pollutants may affect the ecological balance of every ecosystem [2]. Among various ecosystems in the world, rivers which cross different areas such as agriculture and industry are the most threatened and affected by anthropogenic activities [3]. In developing countries such as Iran, water pollution is a common and widespread problem. Therefore, water resource management of rivers is of great importance and especially essential for semiarid and developing countries [4]. Assessment and classification of ecological water quality using indices-based approaches can help the conservation and management of rivers. The measurement of physicochemical parameters is usually time-consuming, cost-intensive and also dependent on special instruments. However, physicochemical parameters can only show water quality at the moment of measurement and these can change over time. Nowadays, indicators based on the presence or absence aquatic organisms have been developed to assess water quality and for the classification of ecological status. Norris and Thoms suggested that the effects on biota are usually the final point of environmental degradation and pollution of rivers and thus are an important indication of ecosystem health [5].

Many living organisms (e.g. small mammals, fish, aquatic plants, algae, invertebrates) can be used to assess ecological water quality. Fish encompass different trophic levels, have a long life cycle, and high mobility, and can herewith be used to integrate the effects of habitat change and environmental pollution over a long period [6]. Macroinvertebrates are used for bioassessment because they are relatively easily sampled and are a very biodiverse group of species inhabiting waters that is contaminated to a different extent (from clean to highly polluted) [7-9]. They are important for the cycling of organic matter and provide food resources for higher trophic levels. The fluctuation of macroinvertebrate richness in the aquatic environment may result in the change of the ecosystem function. Moreover, the relatively low mobility and long life cycles of macroinvertebrates ensure that the presence of a given taxon reflects the past conditions. Many previous studies have shown the importance of biotic indices in the world, especially in Europe [10].

However, there are only a few studies using biotic indices in Asian countries such as Iran. The water quality monitoring programs in Iran are mainly based on the determination of some physicochemical parameters and water quality indices have generally not yet been use as a tool for the assessment and management of river ecosystems. Meanwhile, scientific efforts have often focused on improving freshwater resources that are of economic, cultural or spiritual importance. Unfortunately, most of these efforts have proceeded without documentation of the relative successes and failures of individual activities. Even though success is noted, there is often a lack of biotic data to identify specific results or endpoints for the river management activity [1].

In this study, we tried to employ the most widely used biotic and physicochemical indices to classify the ecological water quality in one of the most important Iranian rivers, the Tajan River. This river was chosen as a pilot river from the 115 rivers in the north of Iran because of having a good water-flow, discharge regime, catchment area, Accessibility and environmental condition [11]. Also, there are many similarities such as environmental landscape, climate and land uses between this river and European rivers. Besides, it is possible to select some stations as references because of being away of human activities.

The goals of this study were to determine and classify the ecological water quality of Tajan River based on different indices of water quality and to evaluate their performance, to zone the water quality based on these indices and GIS (Geographic Information System), to assess the effects of human land uses on the river and to compare the results between up- and down-stream parts of the river. This is the first study that compares biotic and physicochemical indices, as well as uses fish species as a bio indicator in an Iranian River.

## Materials and methods

### Study area

**Figure 1:**
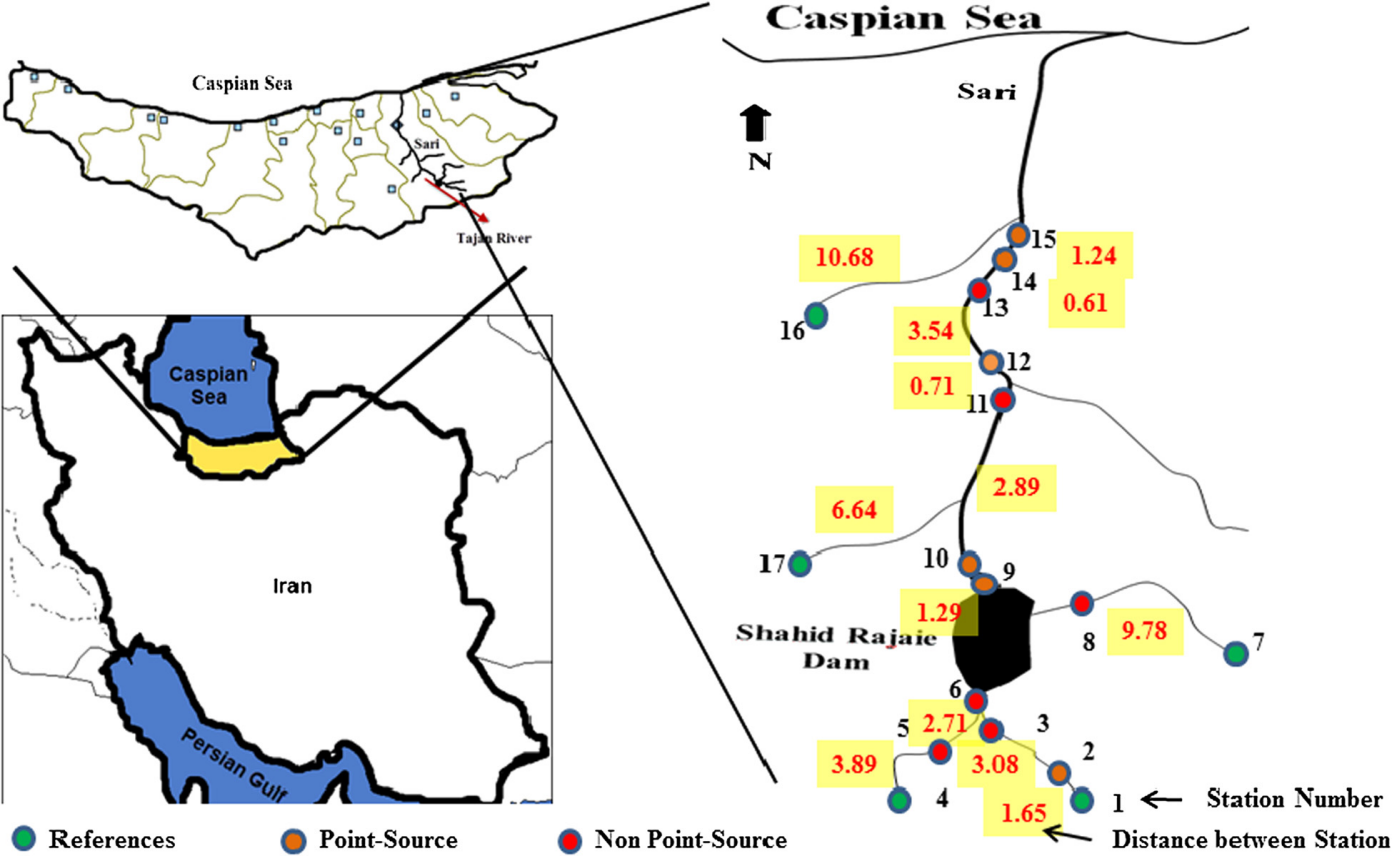
Sites sampled in this case study.

The Tajan River is one of the best freshwater ecosystems in the Mazandaran province located in the north of Iran. The river is 140 km long and originates from forested mountains and continues through different land uses, especially the agricultural areas of the coastal plain, where rice is extensively cultivated. The river eventually terminates in the Caspian Sea, the biggest land-locked aquatic ecosystem in the world (Figure 1). There are different land-uses including agriculture, aquaculture, dam construction, sand mining and industrial activities in the river [11,12]. It is chosen as a pilot river from the 115 rivers in north of Iran [13] because of having a good water-flow, discharge regime, catchment area and environmental condition. The Tajan River is divided to tow parts (Upstream and Downstream) by an old big dam (Shahid-Rajaie Dam, 1993). Water, macroinvertebrates and fish were sampled at 17 sites in September 2013. Eight sites are located in the upstream part and 9 sites in the downstream part. Also, five sites were selected as reference sites where there was no or slight pollution expected. Site selection was based on land use, accessibility and anthropogenic activities. Sites were subjected to non-point (i.e. agricultural runoff) and point influents (i.e. fish pond).

### Physicochemical and habitat parameters

Width, length, depth, altitude (m), dissolved oxygen (mg/l), pH, water and air temperature (°C), conductivity (μS/cm), turbidity (NTU) and nutrients (NO3-N, NO2-N, NH4-N, and PO4-P, mg/l) were measured in situ by using Portable multi-parameter water analyser and UV–vis Spectrophotometry 8000. Biochemical Oxygen Demand (BOD5, mg/l) and Total Suspended Solids (TSS, mg/l) were determined according to APHA [14] with three replicate samples being measured in the laboratory. Some stations in this study overlapped with the stations of Iranian Water Resources Management (IWRM). Therefore, the accuracy of the collected data of physicochemical parameters was checked with the data of IWRM which had collected monthly data for twelve years. Average water flow (m/sec) was calculated by calculating the average of the recordings available from the IWRM for the same period as our sampling. According to the Field Manual for Water Quality Monitoring, the National Sanitation Foundation Water Quality Index (NSFWQI) surveyed 142 sites representing a wide range of locations at the local, state, and national level, using about 35 water quality tests which outcomes were combined in an index [15]. Nine parameters (dissolved oxygen, faecal coliform, biochemical oxygen demand, pH, nitrates, total phosphate, temperature, turbidity, and total solids) were chosen and some were judged to be more important than others, so the values were combined by calculating a weighted mean, based on the method described by Nikoo [16]. For this, field measurements were converted to index values using a questionnaire in which respondents were asked to estimate the level of water quality (0 through 100) corresponding to the field measurements (e.g., pH 2–12). The curves were then averaged and are assumed to represent the best professional judgment of the respondents. When the test results were not available for all 9 measurements, we preserved the relative weight for each factor and scale the total so that the range remains 0 to 100 [17]. The IRWQI_sc_ index is a modification of NSFWQI based on the local condition in Iran. Habitat assessment was performed using 10 factors assessed by four experts and the Rapid Bioassessment Protocol (RBP) was used for river habitat assessment by visual observations at each site [18]. The range of each habitat parameters was from 0 (very perturbed) to 20 (pristine) and included Epifaunal Substrate/Available Cover, Embeddedness (Embed), Velocity/Depth Regime, Sediment Deposition, Channel Flow Status, Channel Alteration, Frequency of Riffles (Freq), Bank Stability, Vegetative Protection, Distance of References (DisRef), and Riparian Vegetative Zone Width. Finally, an average of the ranges of the parameters resulted in an overall RBP index.

### Biological elements

A surber sampler (30*30 cm and 250 micron mesh) was used for collecting macroinvertebrates based on the RBP [18]. Three replicates were collected on each site and all the three replicates were composited as one sample. Benthic macroinvertebrates were preserved in 4% formaldehyde solution before being sorted, identified and counted to family level in laboratory. Electrofishing (200–300 V) with Blank net 6 mm based of FAME Protocol was used to sample fish [19]. Most of the sampled fish were identified, measured and released in situ. Some fish had to be identified further and were fixed in 10% formalin and transferred to the laboratory. Finally, the species and weight of each fish were recorded. RBP metrics were calculated for each sample and only mean site values are reported. In next step, mean values for each site were compared to references sites to generate a score for that site and classification. In Table 1, the classification of introduced indices and their colours are provided [20-25].

**Table 1:** **Indices used to classify water and habitat quality**

	**Range**	**<15**	**15 – 29.9**	**30 – 44.9**	**45 - 55**	**55.1 - 70**	**70.1 - 85**	**85 ≤**
**IRWQI**_**sc**_	Quality	Very Polluted	Polluted	Enough Polluted	Moderate	Enough Good	Good	Excellent
	Colour	Violet	Red	Orange	Yellow	Olive-green	Green	Blue
**NSFWQI**	Range			0-25	25-50	50-70	70-90	90-100
	Quality			Very bad	Bad	Medium	Good	Excellent
**MMIF**	Range			0.00–0.29	0.30–0.49	0.50–0.69	0.70–0.89	0.90–1.00
	Quality			Bad	Poor	Moderate	Good	Excellent
**Karr**	% Index			<40	40-59	60-74	75-87	88-100
	Quality			Very poor	Poor	Fair	Good	Excellent
	Colour			Red	Orange	Yellow	Green	Blue
**RBP**	% Index				<60	74-60	89-75	≥90
	Quality				Poor	Marginal	Good	Excellent
**BMWP** **/ASPT**	Range				4 >	4-5	5-6	6 <
	Quality				Polluted	Moderate	Doubtful	Excellent
	Colour				Red	Yellow	Green	Blue

### Physicochemical and biological indices of water quality

Physical habitat quality (type, stability, availability, etc.) and water quality are reflected by the diversity of stream communities. Based on these relationships, environmental quality of aquatic systems can be described or categorized using integrated approaches that incorporate an evaluation of the physical, chemical and biological components. The guidelines associated with the RBP provide systematic approaches for identifying habitat quality and biotic integrity of river systems [26-28].

The NSFWQI is one of the first water quality indices that aggregate some water quality parameters through a weighted arithmetic mean function [15]. The Index is a usual water quality index method that was developed by paying great rigor in selecting parameters, developing a common scale and assigning weights. The water quality data are recorded and transferred to a weighting curve chart, where a numerical value of Qi is obtained. The mathematical expression for NSF WQI is given by [29]:

$$ \mathrm{NSFWQI}\kern0.5em =\kern0.5em {\displaystyle \sum_{k=1}^n WiQi} $$

Q_i_ = sub-index for each water quality parameter;

W_i_ = weight associated with each water quality parameter;

n = number of water quality parameters.

Also, the Ministry of Energy of Iran developed a local index, IRWQI_sc_ (Iranian Water Quality Index for Surface Water Resource-Conventional Parameters), for the assessment of water quality [23,30]. This index is calculated by:

$$ IRWQI={\displaystyle {\sum}_1^n(IiWi)\raisebox{1ex}{$1$}\!\left/ \!\raisebox{-1ex}{$a$}\right.} $$

$$ a={\displaystyle {\sum}_1^nWi} $$

Ii = sub-index for each water quality parameter;

Wi = weight associated with each water quality parameter;

n = number of water quality parameters;

a = the sum of the weight.

Both indices are based on physicochemical parameters that are evaluated in this study. For assessment and classification of water quality by macroinvertebrate indices, we used the ratio of Biological Monitoring Working Party score to Average Score per Taxon (BMWP/ASPT) and Multimetric Macroinvertebrates Index Flanders (MMIF) [21]. These two indices are numerical expressions used to assess water quality based on the presence and, in many cases, the diversity of taxa with known environmental-pollution tolerances according to the following equation:

$$ \mathrm{B}\mathrm{MWP}/\mathrm{ASPT}\kern0.5em =\kern0.5em \left({\displaystyle \sum \mathrm{B}.\mathrm{n}}\right)/\mathrm{N} $$

Whereas:

B = the value for each species;

n = abundance of each species;

N = total number of species.

Subsequently, ecological water quality is assessed with Karr Biotic Index of Fish (KBI). KBI was firstly invented by Karr to study the River Trent basin in Champaign, then applied by other researchers, and is nowadays used all over the world and well documented by Karr and Ruaro [22,31]. The index is designed to assess the present the status of the community using twelve fish community parameters. These parameters can be roughly grouped into two sets: ecological factors (including number of individuals in samples, the proportion of omnivore individuals, proportion of insectivorous cyprinids, proportion of top carnivorous, proportion of individuals with disease, tumours, fin damage, and other anomalies) and species composition and richness (including number of species, presence of intolerant species, species richness and composition of darters, suckers and sunfish (except green sunfish) and proportion of green sunfish and hybrid individuals) [22]. Habitat condition is classified based on US EPA Rapid Bioassessment Protocol (RBP) [18].

### Statistical analysis

Statistical analysis of the resulting data was performed using Excel and SPSS software version 19 (licensed by Tarbiat Modares University, Iran) and Canoco version 5 (licensed by Wageningen University, The Netherlands). Normality of data was checked by ShapiroWilk test. Because the data were not normally distributed, Mann–Whitney U test were used to assess the significance of the difference between up- and downstream values of physicochemical parameters. Cluster analysis was used for the classification of the stations on the basis of the indices and Casewise analysis was used for assessing the correlation between the classifications (with using SPSS). Principal component analysis (PCA) was used to analyse the correlations between abiotic (habitat and physicochemical) parameters and biotic (macroinvertebrates and fish) community. Before analysis, all the macroinvertebrates were divided into 5 groups based on the pollution tolerance owned by the dominant species in each family (Very sensitive, Sensitive, Neutral, Resistant and Very resistant) that was provided in Appendix B of RBP, EPA [18].

## Results and discussion

For irrigating of the vast plain area of downstream rice farms, the dam valves are opened from spring to late summer and closed in early September, with a minimal flow at all time of 1 m^3^/s. The rains start in early autumn and therefore the best time for sampling is September in accordance with local condition and some previous studies [32]. The main land-use in the upstream areas is not similar to that in the downstream areas, so no similar anthropogenic impact may be expected. Furthermore, there is a larger downstream flow, and herewith also a larger move of pollutants, most of the year because of the opening of the dam valves and irrigation. Habitat parameters show significant differences between up- and downstream sites (see Figure 1 for better understanding). Some land uses such as mining that affect the rivers’ physical status are less development upstream compared to downstream. In the downstream part, there are many mining industries that have altered the physical status of the river. Humans have channelized, diverted, drained and filled streams because of the dredging of sand. In the upstream part of the Tajan River, the higher number of residents has no major impact on the physical structure of habitat. Also, the accessibility of the upstream part of the river is lower than downstreams. Therefore, there are more pristine habitats in upstream. Padmalal et al. showed that in the past 3–4 decades, rivers in the densely populated areas of the world are subjected to immense pressures due to various kinds of human interventions, among which indiscriminating mining for construction-grade sand from alluvial reaches is among the most disastrous one [32].

**Figure 2:**
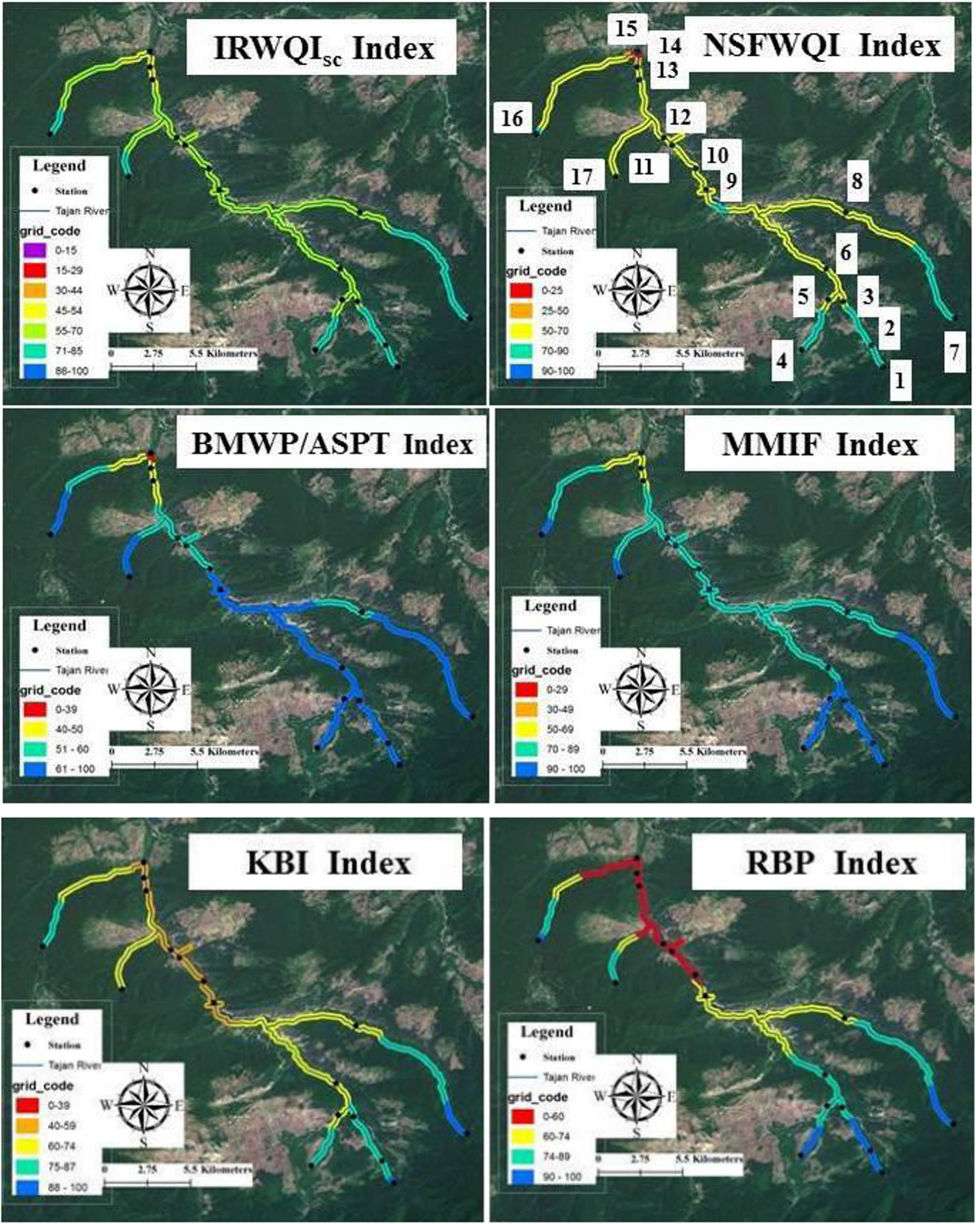
Water quality classification of the Tajan River by different indices and GIS.

We identified 2426 fish individuals covering 17 species, 5 families and 7639 individuals of macroinvertebrates covering 45 geneses or families. Zoning of ecological river condition was performed with GIS based on the result of indices (Table 2 and Figure 2). All indices showed water quality reductions from upstream to downstream: the references stations (1,4,7,16,17) are indicated to have a good water quality (almost unpolluted) and in the most downstream stations, water quality was classified as moderate to polluted (14,15). We tried to get an even distribution of the stations along the river so we could analysis the effects of land uses on study’s parameters, too. Human land use, development and urbanization can alter water flow and degrade stream habitat and biotic conditions through, for example, draining agricultural fields, channelizing streams and increasing sedimentation. The first station is located in an almost pristine area, with hardy any agriculture activities and covered with natural forest. All indices showed that the station was unpolluted. Upstream of the second station, there was a fish pond that probably affected the natural fish community, which is expressed by the low KBI value (green, Figure 2). Other indices, however, did not show a marked response. Because of the limitation to captured fisheries production, aquaculture has been developed worldwide especially in developing countries such as Iran. Aquaculture may have significant impacts on the environment and natural resources, and a number of concerns have been expressed by environmental activists and scientists [33,34]. Raczyńska et al. [35] reported that little attention is paid to the changes in water quality resulting from the inflow of effluent discharged from fish breeding ponds. They, however, report that the aquaculture effluent discharge from the carp breeding ponds had a significant impact on the physicochemical parameters of the river water. They observed an increase in concentrations of the organic and biogenic compounds immediately after the inflow of the polluted water from the ponds [35]. However, more studies are needed in order to understand the decrease in fish biodiversity observed at station 2. Downstream of the fish ponds, rice farms were located. In station 3, physical habitat parameters were altered probably because of the influence of traffic of agriculture. Therefore, the habitat index (RBP) descended from excellent to good levels (Figure 2). Also, the values of macroinvertebrates indices were reduced but no differences were observed for the KBI index. Agriculture may increase nutrient levels, erosion, and turbidity due to the use of fertilizers, planting and harvesting. Especially rice farms may be important for the water quality because they require a lot of water for cultivation. Many scientists studied the effect of agriculture as a non-point pollutant on river basin and biota [36,37]. Traditional agriculture in the research area uses much water and the effluent of the farms often runs directly into the river. The indices did not show many changes for station 6 that is located after of accident tow stream and only the value of the MMIF index decreased. Between station 7 (reference station of the second stretch of the river) and 8, there are large farms and all indices may have decreased in station 8 because of the polluted discharge water from farming. This study is the first one reporting the abundance of fish population in two streams of the Tajan River, as there were 10 fish (*Barbus lacerta*) in station 7. *B. lacerta* is one of the most sensitive species to anthropogenic stress, similar to *Salmo trutta* [38]*. S. trutta* as an indicator species showing a high sensitivity to a variety of human pressures (e.g. water pollution, habitat degradation). Normally, it inhabits headwaters with high oxygen saturation, steep slope, fast flow, suitable temperature regimes and adequate food and due to anthropogenic influences, the brown trout was eliminated from many original habitats in Iran [39]. The comparison of indices in the 8 stations in the upstream catchment showed a comparable decrease in index values between the two streams of the river. A big dam is located between the eight upstream stations and station 9 which changed most of the parameters. Results show that values for pH, TSS, BOD and nutrients were significantly higher in the downstream area compared to the upstream area. The NSFWQI index showed a significant change due to the dam, although the local index (IRWQI_SC_) did not change. This result is similar to other studies on the effect of a dam on physicochemical parameters [40]. The dam altered the diversity and density of macroinvertebrates, but had no effects on the macroinvertebrates indices, because after the dam the total density and total biomass increased, while taxa richness tended to decrease, which is in line with the findings of others [41]. Native fish assemblages have experienced profound changes as a consequence of the dam construction. We documented the decline of native fish and the rise of non-native fish which become the major component of the ichthyofauna in the downstream stations. RBP showed a reduction in the physical structure of the habitat in the downstream part. After the dam, there is a fish pond which may be responsible for the strong reduction in the physical habitat quality as shown by RBP (Table 2). The fish pond are situated very close to the river and also to the dam, and takes in clean water and releases the same amount of effluent water to the downstream part of the river. Downstream of the fish pond there are rice farms and small streams like the Zellem stream entering the main river. In the Zellem stream there were many sand mining and sandblast sites which polluted the water in the Tajan River. The stream is small and is located between station 11 and 12 and a number of indices show a marked decrease between these stations (Table 2). Station 13 and 14 were chosen to show the effect of sand mining which was expressed by difference in levels of IRWQI_SC_. Sand mining increased TSS and negatively affected the physicochemical parameters, herewith affecting some sensitive species such as Salmonids. The most notable changes occurred in this study at station 15 which is located after a wood factory, because the wastewaters of this factory were directly discharged into river. Walker et al. [42] reported that the studies conducted in the early 1990s downstream of pulp and paper mills in Canada, found a number of changes in the physiological, biochemical and reproductive responses of wild fish and macroinvertebrates. The study assessed changes in the taste or odours of fish as a result of exposure to pulp and paper effluent as a measure of effects on the usability of fisheries resources by humans and their conclusion on the effects of the factory on fish and habitat parameters confirms our results [42]. Thompson et al. [43] also described the pulp and paper making industry as the third most water intensive industry in terms of freshwater withdrawal in the world, after the primary metals and the chemical industries. There is a large variation in the quality of the wastewaters from pulping and papermaking operations. This is due to the diversity of processes and the chemicals used [43]. Unfortunately, there has not been any exhaustive study on the effects of the plant’s wastewater on the ecology of the Tajan River in Iran. Effluents of the plant were released without or with a preliminary treatment into the river, and hence the colour and odour of river’s water were changed into brown and strong distinctive odour. There were large differences between station 15 and the others stations, especially the references stations with the same elevation (16, 17). pH and DO decreased and water temperature, BOD and nutrient increased in station 15. The diversity of animals decreased and some exotic species were present (i.e. *Rasborinae pseudorasbora parva*), while the sensitive species were absent. We suggest that more studies on the environmental effects of the paper plant on the ecology of the river are conducted. After station 15, there is a big city, Sari which is the capital of Mazandarn province. A major component of the human use of aquatic systems is the construction, maintenance, and use of roads that occur as part of human infrastructure and the road/stream interface is one of the main pathways for sediment to reach waterways. Stream crossings, often culverts, can alter in-stream sediment accumulation and the geomorphology of a stream. The effects of sedimentation on macroinvertebrates and fish have been well documented [1], as well as of traffic, excavating bottom sand from the river and changing of the riparian zone , which likely caused the obviously decrease in the habitat parameters at the station.

**Table 2:** **The value of the calculated indices for each station**

**Station**	**Longitude**	**Latitude**	**KBI**	**BMWP/ASPT**	**MMIF**	**NSFWQI**	**IRWQIsc**	**RBP**
**1**	712608	4004824	99	8.9	0.95	81.49	87.99	100
**2**	712059	4005260	75.03	7.5	0.95	70.91	74.06	95
**3**	709238	4006751	76.28	6.6	0.95	66.97	68.39	88.5
**4**	708189	4001145	81.48	8.2	0.95	81.74	81.26	100
**5**	707769	4006595	69.24	6.7	0.95	61.62	62.57	82
**6**	706166	4010260	75.94	5.7	0.85	59.6	54.84	79
**7**	726606	4007091	100	7.8	1	69.37	89.39	97
**8**	707556	4013824	70.95	5.8	0.85	63.59	72.06	73
**9**	700152	4014338	49.05	7.8	0.8	67.95	80.29	61
**10**	699857	4015151	52.03	5.2	0.8	59.67	62.2	58.5
**11**	695882	4025071	56.21	5.3	0.75	59.72	64.38	57.5
**12**	695429	4026319	51.96	5.5	0.7	52.02	52.36	44.5
**13**	689155	4037624	60.86	4.2	0.7	52.47	50.9	46.5
**14**	688694	4038040	52.08	4.2	0.65	53	46.82	38
**15**	686889	4039978	56.61	3.6	0.55	44.51	39.16	34.5
**16**	685112	4033640	90.53	7.6	0.95	67.84	86.57	96
**17**	689592	4023526	83.46	8.2	0.95	66.14	88.64	97

**Figure 3:**
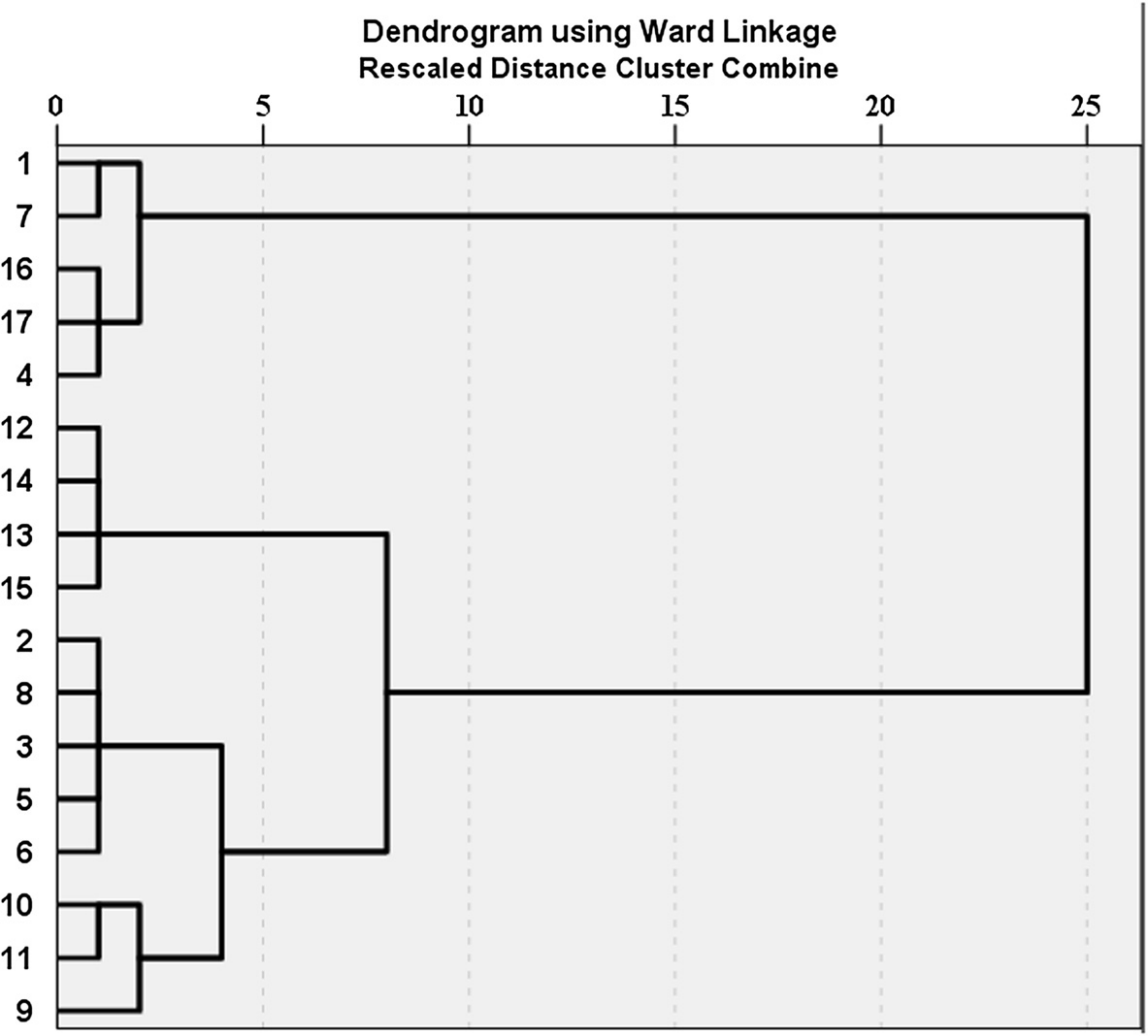
Classification of stations on based biotic indices.

**Figure 4:**
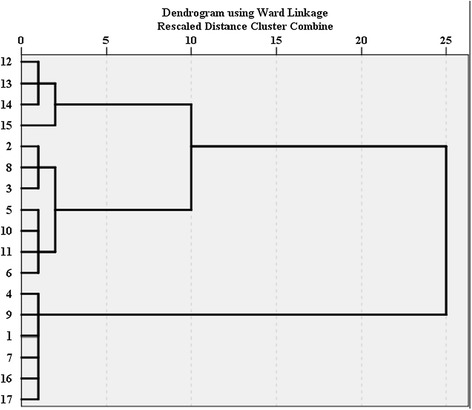
Classification of stations on based abiotic indices.

Figure 3 shows the classification of the stations of the Tajan River based on biotic indices (KBI, MMIF and BMWP/ASPT) and Figure 4 shows it based on abiotic indices (IRWQIsc, NSFWQI). The dendrogram of the biotic indices divides all stations into two major groups. All references stations are present in the first group and others stations are in the other group. The stations 12, 13, 14 and 15 are then subdivided in a separate group and these stations were located downstream with heavy human activities (Figure 3). The classification based on the abiotic indices shows the impact of land uses on river ecosystems very well. The classification groups the references stations together with station 9 which is located after dam because the dam improved the water quality by reducing sediment deposition, temperature go down and other physicochemical parameters partly improve. The results indicate also the percentage of group membership. On the basis of the results, KBI (fish index) could predict 61% of the changes in physicochemical parameters using our sampling data and 94% of these changes when using the 12 years of data. The prediction ability based on macroinvertebrates indices (MMIF and BMWP/ASPT) were 76 and 71% for our sampling data and 12 years of data, respectively. Fish are at the top of the food chain and could, therefore, show the effects of differences in water pollution at longer time scales than macroinvertebrates, explaining the higher predictive value of the fish index compared to the macroinvertebrate indices. Macroinvertebrate indices had a better correlation with physicochemical parameters in our sampling time, compared to the fish one, probably because of their shorter life cycle, greater dispersal ability, and lower position in the food chain than fish.

**Figure 5:**
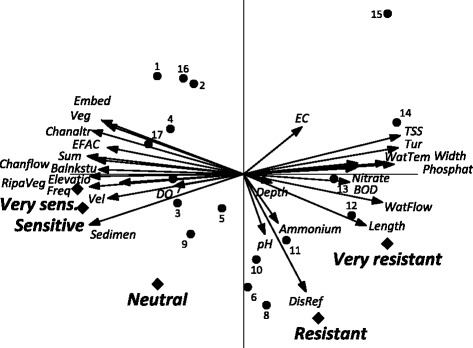
The correlations between abiotic parameters and macroinvertebrates sensitivity groups (PCA, Canoco 5). Of the variation in group abundances 75% is displayed on the horizontal axis and another 17% on the vertical one.

With the increase of the habitat parameters located at the left side of the diagram, the abundance of the very sensitive and sensitive fish species also increase (Figure 5). DO is the only physicochemical parameters that has a positive correlation with the abundance of the very sensitive and sensitive species. The other physicochemical parameters have negative correlations with both groups (Figure 5). Also the stations with a good status, such as 9, are located on the left side of the diagram. The arrow of neutral species indicates that this group was not strongly correlated with any of the parameters. In other words, the abundance of this group is not correlated with pollution and cannot be used as an indicator. On the right side there are the polluted stations and the groups of resistant and very resistant species. The arrows of the resistant species, pH and Ammonium point into the same direction, which means these two parameters correlate with the abundance of resistant species and less with the others parameters. The abundance of the very resistant species increases with most of the studied physicochemical parameters (except DO) and decrease of habitats parameters. In the upper right quadrant the highly polluted stations are located, with the placement of station 15 being extreme because it receives high pollution from a pulp and wood plant. The result of the PCA analysis complements was similar to the result of bio indicators and classification of stations.

**Figure 6:**
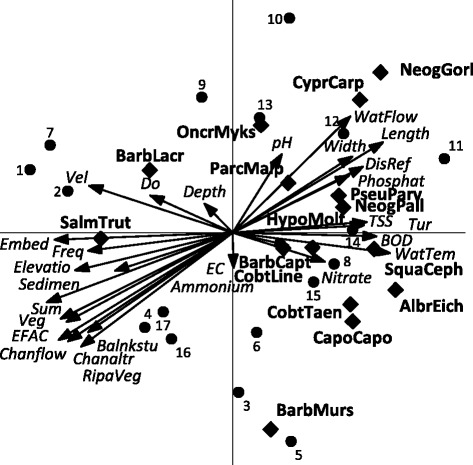
The relationship between abiotic parameters and fish species (PCA, Canoco 5). Of the variation in group abundances 31% is displayed on the horizontal axis and another 23% on the vertical one.

Also, the result of the PCA analysis on the fish data and abiotic parameters shows that the placement of two fish species (i.e. *B. lacerta* and *S. trutta*) are different from the others (Figure 6). *B. lacerta* and *S. trutta* are correlate positively with high DO and habitat related parameters, while the opposite is true for most other species (Figure 6). On the other hand, the two species are indicated to have a negative correlation with most polluted related physicochemical parameters and therefore may be suitable to indicate clean water. As the physicochemical parameters except DO increase, the presence of the sensitive species decrease which is in accordance with Bengar et al. [44]. Habitat related parameters and DO had a negative correlation with most of the other fish species in the Tajan River and most fish species increase in abundance in the stations at the right side of the diagram (such as station 11, 12).

## Conclusion

This study provides an assessment and comparison of biotic and abiotic indices-based approaches for the Tajan River. As well as Yazdian et al. concluded, we cannot claim that all of the indices would work in other regions as well [45], because of the different range in biodiversity, physicochemical parameters and land-uses. Actually, we have to use, modify and develop some native indices for Iranian ecosystems that they can be used for a rapid assessment of environmental health condition. A comparison between the indices shows that the classification based on biotic indices can show the long-term environmental condition better than those based on abiotic indices. We suggest that macroinvertebrates and fish can be used as indicators of water pollution with having the advantages of low cost, easy identification and it provides a better reflection of water quality than using physicochemical parameters alone. We also suggest to develop and modify the biotic indices for research and management of Iranian rivers, since Iran is located in the mid-dry area where water resources management is particularly urgent and important.
